# The Cytoreductive Effect of Radiotherapy for Small Cell Ovarian Carcinoma of the Pulmonary Type: A Case Report and Review of the Literature

**DOI:** 10.1155/2018/4383216

**Published:** 2018-01-16

**Authors:** Shuhei Terada, Takashi Suzuki, Akihiro Hasegawa, Satoru Nakayama, Hiroshi Adachi

**Affiliations:** Department of Gynecology, Seirei Hamamatsu General Hospital, 2-12-12 Sumiyoshi, Naka-ku, Hamamatsu, Shizuoka 430-8558, Japan

## Abstract

Small cell ovarian carcinoma of the pulmonary type is a rare and highly aggressive tumor for which a suitable treatment strategy has not been established. A 45-year-old woman presented with abdominal swelling, and primary ovarian carcinoma was suspected. The postoperative pathological diagnosis was small cell ovarian carcinoma of the pulmonary type. She also had complicated grade 1 endometrioid carcinoma of the uterine corpus. Three courses of cisplatin and etoposide therapy were administered as adjuvant chemotherapy. Because the tumor was chemotherapy resistant, she underwent palliative abdominal irradiation at a dose of 26 Gy in 13 fractions, which induced cytoreduction and provided symptomatic relief. She died 4 months after surgery. Lactate dehydrogenase was a useful tumor marker during treatment. Here, we present an extremely rare case of a patient with small cell ovarian carcinoma of the pulmonary type treated with radiotherapy after surgery and chemotherapy.

## 1. Introduction

Small cell ovarian carcinoma pulmonary type (SCOCPT) is defined as a small cell carcinoma that resembles a neuroendocrine type of pulmonary small cell carcinoma. Most patients with SCOCPT are postmenopausal and present with symptoms such as a pelvic or abdominal mass. SCOCPTs are highly aggressive neoplasms, which usually present at an advanced stage, and the overall prognosis is poor. Only 26 cases of SCOCPT have been reported in the literature, and most patients have typically been treated with surgery followed by chemotherapy. Palliative brain irradiation was administered to control seizures in the previous report [1], but the cytoreductive effect of radiotherapy on the primary lesions is unknown. Here we present a case in which a patient with chemotherapy-resistant SCOCPT underwent abdominal radiotherapy. We describe the outcomes of the radiotherapy and also present evidence on lactate dehydrogenase (LDH) potentially acting as a good tumor marker during the course of treatment.

## 2. Case Presentation

**Figure 1:**
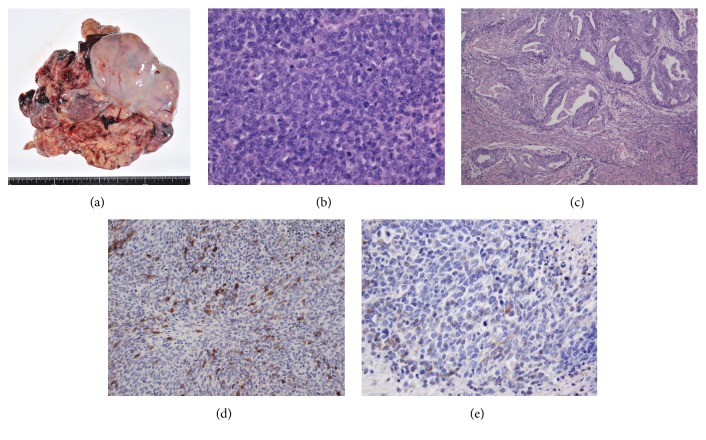
(a) Macroscopic findings: an irregular, white, and solid tumor of the right ovary. ((b)–(d)) Microscopic findings. (b) Tumor cells are round to ovoid with hyperchromatic nuclei, scant cytoplasm, abundant mitosis, and apoptosis with conspicuous necrosis (hematoxylin-eosin stain (HE), ×300). (c) Endometrial carcinoma grade 1 in uterine corpus (HE, ×30) with less than half myometrial invasion and no vascular invasion. ((d), (e)) Tumor cells are positive for synaptophysin and neuron-specific enolase (immunostaining, ×300).

A 45-year-old woman (gravida 1, para 1) presented at our hospital with abdominal swelling. She had undergone laser ablation of cervical intraepithelial neoplasia 3 (CIN3) 12 years previously. She had a family history of paternal prostate cancer. Physical examination showed a large solid tumor in her pelvic region. Abdominal magnetic resonance imaging (MRI) identified a unilateral ovarian mass and small ascites. The tumor, approximately 12 cm in diameter, revealed a mixed pattern of multicystic and solid parts. Uterine endometrial thickening was also observed, and endometrial biopsy revealed grade 1 endometrioid carcinoma. Computed tomography (CT) revealed no lymph node metastasis or extrapelvic tumors. The laboratory data showed an elevated level of LDH (1246 U/L, normal < 210 U/L). Isozyme analysis results were as follows: LD1 15%, LD2 30%, LD3 27%, LD4 18%, and LD5 10%. The serum level of cancer antigen 125 (CA125) was 156.7 U/mL (normal < 35 U/mL). The serum calcium level was normal. Double cancer of the ovary and endometrium was suspected, and a staging laparotomy was scheduled. Eighteen days after first consultation, the patient complained of severe abdominal pain, and ultrasonography revealed that the tumor had enlarged to 16 cm in diameter, with an accumulation of ascitic fluid and newly diagnosed peritoneal dissemination. The tumor was rapidly progressing, and tumor rupture was also suspected. She underwent an urgent laparotomy, which revealed that a white and solid tumor of the right ovary had spontaneously ruptured in the intra-abdominal cavity (Figure 1(a)). There was massive bloody ascites of approximately 2700 mL in volume. The tumor was strongly adherent to the bladder and sigmoid colon and was difficult to decorticate. Multiple, but small, peritoneal disseminations were observed in the pelvic cavity. Total abdominal hysterectomy, bilateral salpingo-oophorectomy, and partial omentectomy were performed, and a 5 × 4 cm tumor remained behind the bladder due to the strong adhesion. The pathological diagnosis was SCOCPT (Figure 1(b)) and grade 1 endometrioid carcinoma of the uterine corpus (Figure 1(c)). Immunostaining of the ovarian tumor was positive for epithelial membrane antigen, synaptophysin (Figure 1(d)), neuron-specific enolase (NSE) (Figure 1(e)), p16, and cluster of differentiation (CD) 56 and negative for cytokeratin 20, Wilms Tumor 1, thyroid transcription factor 1, and anti-cytokeratin (CK) antibodies CK18, CK19, and CD99. Thus, we diagnosed primary ovarian cancer stage IIIC (pT3cNxM0) and endometrial cancer stage IA (pT1aNxM0) according to the International Federation of Gynecology and Obstetrics classification system. The serum level of NSE after surgery was 28.1 ng/mL (normal < 16.3 ng/mL).

**Figure 2:**
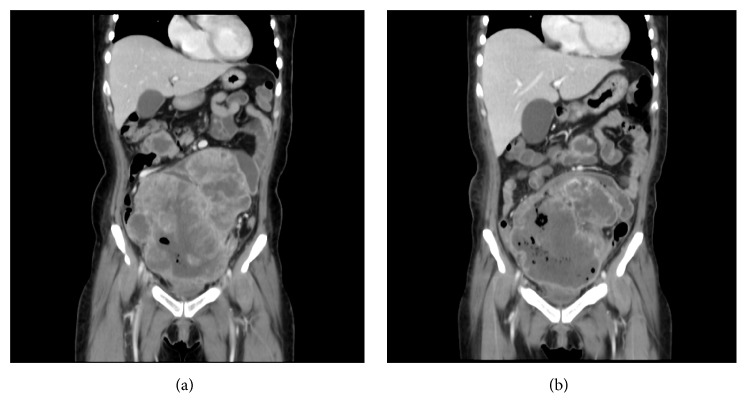
(a) Enhanced abdominal computed tomography (coronal section) reveals the remaining tumor is approximately 20.3 cm in diameter before irradiation. (b) After radiation therapy, the tumor size decreased to 15.3 cm.

**Figure 3:**
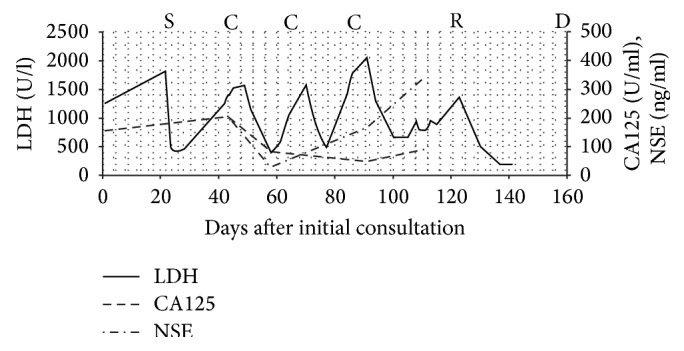
The graph shows the time course of the levels of serum lactate dehydrogenase (LDH), cancer antigen 125 (CA125), and neuron-specific enolase (NSE). The patient underwent surgery (S) on day 21; chemotherapy (C) on days 43, 65, and 86; and abdominal irradiation (R) from day 120 to day 140. She died on day 157 (D). The solid line shows levels of LDH, the dashed-line shows levels of CA125, and the dashed-dotted line shows levels of NSE.

Twenty days after surgery, a CT scan revealed that the remaining tumor in the pelvic cavity had enlarged to 14.8 cm. At 21 days after surgery, the patient received adjuvant chemotherapy combining cisplatin (60 mg/m^2^ on day 1) and etoposide (100 mg/m^2^ on days 1 to 3) administered every 3 weeks by intravenous infusion. After each course, CT scanning revealed slight shrinkage of the residual tumor, but it enlarged during the drug holidays. After 3 courses of this regimen, the tumor was observed to have enlarged to 20.3 cm on CT. The patient's performance status, as measured using the Eastern Cooperative Oncology Group (ECOG) grading scale, was grade 3, and she complained of abdominal swelling. Therefore, irradiation of the abdominal tumor was initiated. A total dose of 26 Gy was given in 13 fractions, and the tumor size decreased to 15.3 cm with improved symptoms (Figure 2). However, several hypointensity lesions suggestive of intratumor gas were observed in the pelvic tumor. The patient gradually felt severely fatigued and refused any treatment. She was transferred to a hospice and died 135 days after the surgery. An autopsy was not performed. During the course of treatment, serum LDH levels repeatedly decreased soon after the chemotherapy but increased during drug holidays (Figure 3).

## 3. Discussion

We identified two important clinical issues. Palliative radiotherapy for SCOCPT can be considered in patients with abdominal swelling, and LDH is a useful tumor marker.

First, with regard to palliative radiotherapy as a cytoreductive effect for SCOCPT, a total of 26 cases of SCOCPT have been reported in the literature, and 18 of these were treated with chemotherapy postoperatively (Table 1) [1–15]. To our knowledge, this is the first report of SCOCPT treated with radiotherapy except for brain irradiation to control seizures with brain metastasis. Palliative radiotherapy at a dose of 26 Gy for the residual abdominal tumor contributed to tumor shrinkage by approximately 25%. Intratumor gas was observed, but it was not ascertained that the fistula between the tumor and the intestines was attributable to radiotherapy. It is not clear if radiotherapy improved the prognosis. However, we believe that radiotherapy was at least effective for improving the symptoms associated with tumor enlargement.

There are many reports regarding the effectiveness of radiotherapy in small cell carcinoma in other organs. Radiotherapy plays a vital role in the management of the full spectrum of small cell lung cancer, from its ability to palliate symptoms to improvements in survival [16]. Thoracic radiotherapy combined with chemotherapy improves survival in patients with limited-stage small cell lung cancer [17, 18]. According to the National Comprehensive Cancer Network guideline, concurrent chemoradiotherapy (CCRT) is recommended for limited-stage small cell lung cancer in excess of T1-2 N0 [19]. According to reports on small cell carcinoma in other sex organs, including the uterine cervix, endometrium, and vagina, multimodality therapy is likely the treatment of choice [20]. Patients with SCOCPT never received pelvic or abdominal radiation in previous reports. It is reasonable to follow treatment trends for small cell carcinoma of the lung, since tumors are histologically indistinguishable and exhibit a similar aggressive behavior.

Second, with regard to LDH as a useful tumor marker of SCOCPT, CA125 (positive rate 82.4%), NSE (85.7%), and CA19-9 (37.5%) have been reported as tumor markers for SCOCPT [21], but LDH has not been reported. Increased LDH is a prognostic tumor marker in many other solid tumors, including non-small cell lung cancer [22], breast cancer [23], and prostate cancer [24]. It has long been known that many human cancers have higher LDH levels than normal tissues [25]. The serum level of LDH is thought to be correlated with the tumor burden and to reflect the tumor's growth and invasive potential [26]. In this case, chemotherapy and radiotherapy transiently inhibited the growth of both tumor cells and LDH production, which led to an acute decrease in the serum LDH level. Because of the highly proliferative capacity of SCOCPT, serum LDH levels increased during the drug holidays. Although LDH has five isoforms with different distributions, it was not necessary to detect these isoforms separately because no specific isoform was characteristically increased. This is useful, because total serum LDH assessment is convenient and inexpensive [27]. Serum LDH can be a useful tumor marker to evaluate the effectiveness of the treatment and to assess the prognosis.

## 4. Conclusion

Palliative radiotherapy for SCOCPT can be considered in patients with abdominal swelling, and LDH might be a useful tumor marker. Radiotherapy might be considered as an option if the tumor is resistant to chemotherapy after a staging laparotomy. Furthermore, multimodality therapy might be appropriate in patients thought to be candidates for aggressive, potentially curative treatments, but further research is required to establish the treatment strategy for SCOCPT.

## Figures and Tables

**Table 1:** A review of the literature: the characteristics, the treatment, and the outcome of small cell ovarian carcinoma, pulmonary type.

Author	Year	Age	Stage	Operation	Postoperative treatment	Outcome
Eichhorn et al. [1]	1992	62	1a	RSO	None	DOD, 4 Mo
		59	1a	TAH, RSO	Unknown	Unknown
		55	1a	TAH, BSO	Unknown	Unknown
		28	1c	TAH, BSO, OMT, appendectomy, peritoneal biopsies	Unknown	AWD, 6 Mo
		85	2b	BSO	None	DOD, 1 Mo
		76	3b	BSO (subtotal resection)	Unknown	DOD, 12 Mo
		50	3b	LSO	Unknown	Unknown
		72	3b	TAH, BSO, OMT	CDDP, CPM	DOD, 12 Mo
		64	3b	TAH, BSO, OMT, LND, colectomy, appendectomy	CDDP, CPM, ADR	AWD, 8 Mo
		49	3b	LSO, ileocolectomy, right para-aortic LND	CDDP, CPM, ADR, MTX, 5-FU	DOD, 13 Mo
		46	3c	TAH, BSO, retroperitoneal LND	CDDP, CPM, ADR, VP-16, VCR	ANED, 7.5 years

Chang et al. [2]	1992	22	1a	LSO, enucleation of the right ovarian tumor	CDDP, CPM, ADR	ANED, 84 Mo

Fukunaga et al. [3]	1997	64	1a	TAH, BSO, OMT, pelvic and para-aortic LND	CDDP, CPM	DOD, 10 Mo

Lim et al. [4]	1998	28	4b	TAH, BSO	CDDP, VP-16, BLM	ANED, 34 Mo

Mebis et al. [5]	2004	54	3c	TAH, BSO, OMT	CDDP, IFM, VP-16	DOD, 14 Mo

Rund and Fischer [6]	2006	56	3c	TAH, BSO, OMT, pelvic LND, splenectomy, segmental resection of ileum and sigmoid colon	CDDP, VP-16	AWD, 7 Mo
	2006	39	3a	TAH, BSO, OMT	CDDP, VP-16, PTX	DOD, 16 Mo

Grandjean et al. [7]	2007	32	1a	BSO, OMT, left pelvic LND	CDDP, VP-16	ANED

Saitoh-Sekiguchi et al. [8]	2007	55	3c	TAH, BSO, OMT, cytoreductive surgery of dissemination	CDDP, CPT-11	ANED, 12 Mo

Suzuki et al. [9]	2007	49	1c(a)	TAH, BSO, pelvic and para-aortic LND, OMT	CBDCA, PTX	ANED, 36 Mo

Reckova et al. [10]	2010	67	4b	TAH, BSO, OMT, appendectomy	CBDCA, VP-16	DOD, 24 Mo

Ikota et al. [11]	2012	68	1a	TAH, BSO, OMT	None	ANED, 10 Mo

Kira et al. [12]	2012	33	3c	TAH, BSO, OMT, pelvic and para-aortic LND	CDDP, CPT-11	DOD, 6 Mo

Tsolakidis et al. [13]	2012	55	3c	TAH, BSO, OMT, sigmoidectomy, pelvic and para-aortic LND	CBDCA, VP-16	AEND, 21 Mo

Kurasaki et al. [14]	2013	54	3a	TAH, BSO, OMT	CBDCA, PTX	ANED, 22 Mo

Rubio et al. [15]	2015	37	3b	TAH, BSO, OMT, pelvic LND	CDDP, VP-16	DOD, 4 Mo

TAH, total abdominal hysterectomy; BSO, bilateral salpingo-oophorectomy; LND, lymph node dissection; OMT, omentectomy; CDDP, cisplatin; CBDCA, carboplatin; PTX, paclitaxel; ADR, doxorubicin; CPM, cyclophosphamide; VP-16, etoposide; CPT-11, irinotecan; IFM, ifosfamide; BLM, bleomycin; VCR, vincristine; MTX, methotrexate; 5-FU, 5-fluorouracil; DOD, dead of disease; ANED, alive with no evidence of disease; AWD, alive with recurrent or residual disease; Mo, months.
